# Incidence trends and risk factors for hyponatremia in epilepsy patients: A large-scale real-world data study

**DOI:** 10.1016/j.heliyon.2023.e18721

**Published:** 2023-07-26

**Authors:** Yoshiaki Yamamoto, Akiko Ohta, Naotaka Usui, Katsumi Imai, Yoshiyuki Kagawa, Yukitoshi Takahashi

**Affiliations:** a Department of Clinical Research, NHO, National Epilepsy Center, Shizuoka Institute of Epilepsy and Neurological Disorders, 886 Urushiyama, Shizuoka, 420-8688, Japan; b Department of Clinical Pharmaceutics, Graduate School of Pharmaceutical Sciences, University of Shizuoka, 52-1 Yada, Shizuoka, 422-8526, Japan; c Laboratory of Clinical Pharmacokinetics and Drug Safety, Shizuoka General Hospital, 4-27-1 Kita Ando, Shizuoka, 420-8527, Japan

**Keywords:** Hyponatremia, Pediatrics, Epilepsy, Polypharmacy, Adverse events

## Abstract

**Objective:**

This study aimed to evaluate the annual incidence and risk factors of hyponatremia in pediatric, adult, and older adult patients with epilepsy.

**Methods:**

We enrolled 26,179 patients: 8598 pediatric patients (aged 0–15 years), 16,476 adults (aged 16–64 years), and 1105 older adults (aged ≥65 years). Patients were included if their serum sodium levels were measured between January 2006 and December 2020. Moderate-severe hyponatremia was defined as a serum sodium level of less than 130 mEq/L.

**Results:**

From 2006 to 2020, 677 patients (2.6%) developed moderate-severe hyponatremia. The incidence of hyponatremia per 1000 person-years was 3.1 in the pediatric group, 19.8 in the adult group, and 50.4 in the older adult group. The incidence increased markedly from 36.8 in 2007 to 58.5 in 2020 in the older adult group but remained unchanged in the adult group and tended to decrease in the pediatric group. In the multiple logistic regression model, use of carbamazepine, valproate, phenytoin, phenobarbital, benzodiazepines, and antipsychotics was found to be a significant risk factor for hyponatremia. In adult patients, carbamazepine, benzodiazepine, and antipsychotics induced hyponatremia in a dose-dependent manner. Concomitant use of zonisamide reduced the risk of hyponatremia.

**Significance:**

Hyponatremia will become an increasingly important concern in clinical settings because the population of epilepsy patients is aging. Serum sodium levels should be monitored carefully when patients are receiving first-generation antiseizure medications or antipsychotics or combinations of these drugs. Our findings may help to minimize the risk of hyponatremia in epilepsy patients.

## 1 Introduction

Hyponatremia is a frequent electrolyte disorder in clinical settings and occurs in up to 30% of hospital patients. Generally, hyponatremia is defined as a serum sodium level of less than 135 mEq/L and is subdivided into the following grades: mild (130–134 mEq/L), moderate (125–129 mEq/L), and severe (<125 mEq/L) [1].

Hyponatremia is an additional mortality risk factor in inpatients and is associated with an increase in the length of the hospital stay and utilization of hospital resources. Winzeler et al. conducted a meta-analysis and showed that improvement of hyponatremia is independently associated with a reduction of mortality risk [2]. Halawa et al. reported that hyponatremia is associated with epileptic seizures [3]: The odds ratio for the development of seizures was 3.9-fold higher in patients with a serum sodium level of 115–119 mEq/L than in those with a serum sodium level of 120–124 mEq/L. Several researchers have studied hyponatremia caused by antiseizure medications (ASM), and they defined the cutoff value as less than 135 mEq/L [[4], [5], [6], [7], [8], [9], [10]]. Other studies used hospitalization for hyponatremia as a clinical endpoint [11,12]. Among the ASMs, carbamazepine, oxcarbazepine, and eslicarbazepine may be associated with a reduction of the serum sodium level [7,8,11,12]. In addition, several studies suggested that the incident risk of hyponatremia increases with increasing carbamazepine dose or serum level [9,13,14]. Thus, serum sodium levels must be carefully monitored in epilepsy patients to prevent adverse events. The mechanism of carbamazepine-related hyponatremia is considered to be hypersecretion of arginine vasopressin from the posterior pituitary; however, an experimental study indicated that carbamazepine has a direct effect on the kidney through vasopressin V2 receptor stimulation without increasing the release of endogenous arginine vasopressin [10,15].

In contrast, it is unclear whether hyponatremia is associated with other ASMs, including valproate, lamotrigine, and levetiracetam [[16], [17], [18]]. Although the cited reports suggest that these ASMs have an increased risk of hyponatremia, they were case reports or case series studies of several patients and not randomized controlled trials or retrospective studies of large databases.

Previously, we evaluated 14,620 adult epilepsy patients and found that concomitant use of phenobarbital, benzodiazepines, and antipsychotics are risk factors for hyponatremia [19]. Moreover, Falhammar et al. conducted a population-based case-control study and showed that newly initiated treatment with levetiracetam is strongly associated with hospitalization for hyponatremia [12].

Earlier studies analyzed risk factors of hyponatremia in adults or older adults, and little research has been published on pediatric patients. In addition, it is unclear whether the third-generation ASMs perampanel and lacosamide affect serum sodium levels. Therefore, we conducted additional analyses to further evaluate the incidence trends and risk factors for hyponatremia in pediatric, adult, and older adult epilepsy patients.

## 2 Methods

### 2.1 Participants

We enrolled 26,179 patients in whom the serum sodium level was measured between January 2006 and December 2020 at the National Epilepsy Center, Shizuoka, Japan. This study included 14,620 adult patients who were enrolled in a previous study (study period: January 2006 to December 2017) [19]. Serum sodium levels were analyzed by using a VITROS5600 autoanalyzer (Ortho Clinical Diagnostics, Tokyo, Japan). Our hospital is a tertiary care epilepsy center in Japan. The majority of patients enrolled in this study had refractory epilepsy.

We retrospectively reviewed the electronic clinical records of these patients and investigated the following information: age, sex, body weight, clinical symptoms, concomitant ASM treatment, ASM dose and concentration, and other laboratory data. During the study period, we assayed 312,262 serum samples. The serum sodium level was measured multiple times in most patients. If a patient's ASM regimen was changed during the study period, we used the sample with the lowest sodium level.

Moderate-severe hyponatremia was defined as a serum sodium level of less than 130 mEq/L by the same method as used in our previous study (in accordance with the Common Terminology Criteria for Adverse Events; 1 mEq/L = 1 mmol/L).

We excluded patients who received diuretics, anti-cancer agents, or immunosuppressants; patients with a severe infection (C-reactive protein >10 mg/dL); and patients with severe hepatic or renal dysfunction (aspartate aminotransferase or alanine aminotransferase >300 U/L and/or serum creatinine >3.0 mg/dL). Also, we excluded measurements made within 4 weeks of patients starting or adding an ASM. None of the patients received oxcarbazepine or eslicarbazepine because these ASMs are not approved in Japan.

Primidone is converted by the liver to phenobarbital, so it was considered to be equivalent to phenobarbital. To assess whether the risk of hyponatremia was associated with the dose of primidone, we converted the daily dose of primidone into phenobarbital equivalents by using the defined daily dose (DDD, WHO Collaborating Centre for Drug Statistics Methodology, DDD Index 2021. Available from: https://www.whocc.no/atc_ddd_index/). Similarly, the doses of benzodiazepines and antipsychotics were transformed into doses of diazepam and chlorpromazine, respectively [20].

The study protocol was approved by the ethics committee of our hospital, patients gave general consent (opt-out agreements).

### 2.2 Statistical analysis

For data analysis, we divided the patients into three age groups: pediatric patients, aged 0–15 years; adult patients, aged 16–64 years; and older adult patients, aged 65 years or older. First, we explored potential risk factors for hyponatremia in each group by univariate analysis. To compare the incidence rate between patients with and without hyponatremia, we performed a chi-square test. We used any factors with a significant influence on the development of hyponatremia (*P* < 0.05), age, and sex as the independent variables (covariates). To assess the risk of hyponatremia, we used logistic regression analysis to calculate crude odds ratios with 95% confidence intervals and multiple logistic regression analysis to calculate adjusted odds ratios with 95% confidence intervals.

All results are expressed as means or odds ratios with 95% confidence intervals. Statistical analysis was performed with SPSS software Ver 25.0 (IBM Corp., Armonk, NY, USA).

## 3 Results

### 3.1 Patient profile

Table 1 shows the clinical characteristics of the three age groups. The pediatric group comprised 435 infants (age, <1 year; 5.1%), 2941 preschool children (age, 1–5 years; 34.2%), 3124 primary school children (age, 6–11 years; 36.3%), and 2098 adolescents (age 12–15 years, 24.4%). The rate of concomitant ASM use differed between the pediatric, adult, and older adult patients. In particular, pediatric patients had a low rate of concomitant use of carbamazepine, phenytoin, and phenobarbital, and older adults had a low rate of concomitant use of valproate, zonisamide, topiramate, and benzodiazepine use. The highest rate of antipsychotic drug use was in the adult group. The overall cohort also included patients who were not taking ASMs; these patients had newly diagnosed epilepsy, suspected epilepsy, or seizures that did not signify epilepsy or had completed ASM therapy. We used this group as a control to evaluate the influence of ASMs on the risk of hyponatremia. Among the patients treated with antipsychotics, 391 received first-generation antipsychotics (e.g., chlorpromazine and haloperidol); 822, second-generation antipsychotics (e.g., risperidone and aripiprazole); and 166, a combination of first- and second-generation antipsychotics.

**Table 1:** Participant characteristics.

	Pediatric patients (0–15 years)	Adult patients (16–64 years)	Older adult patients (≥65 years)
Total number of patients	8598	16,476	1105
Age, mean (95% CI), y	7.4 (7.3–7.5)	33.0 (32.8–33.2)	72.5 (72.1–72.9)
Sex, n (%), female	3687 (42.9)	7619 (46.2)	480 (43.4)
Concomitant drugs, n (%)			
No ASMs, n (%)	2459 (28.6)	2755 (16.7)	312 (28.2)
Mean (95% CI)	1.39 (1.36–1.41)	1.68 (1.66–1.70)	1.23 (1.16–1.29)
Monotherapy, n (%)	2408 (28.0)	5613 (34.1)	409 (37.0)
Polytherapy, n (%)	3731 (43.4)	8108 (49.2)	384 (34.8)
Valproate, n (%)	3433 (39.9)	6106 (37.1)	216 (19.5)
Carbamazepine, n (%)	1681 (19.6)	5228 (31.7)	303 (27.4)
Phenytoin, n (%)	444 (5.2)	3064 (18.6)	218 (19.7)
Phenobarbital, n (%)	629 (7.3)	2643 (16.0)	186 (16.8)
Zonisamide, n (%)	1136 (13.2)	1541 (9.4)	47 (4.3)
Benzodiazepines, n (%)	1548 (18.0)	3469 (21.1)	119 (10.8)
Lamotrigine, n (%)	759 (8.8)	1647 (10.0)	45 (4.1)
Levetiracetam, n (%)	1132 (13.2)	1952 (11.8)	120 (10.9)
Topiramate, n (%)	444 (5.2)	457 (2.8)	7 (0.6)
Lacosamide, n (%)	100 (1.2)	376 (2.3)	58 (5.2)
Perampanel, n (%)	81 (0.9)	354 (2.1)	12 (1.1)
Antipsychotics (%)	161 (1.9)	1185 (7.2)	33 (3.0)
Serum sodium level, mean (95%CI), mEq/L^∗^	139.1 (139.1–139.2)	138.8 (138.8–138.9)^†^	138.5 (138.2–138.8)^†,‡^
Hyponatremia			
Grade 1 (<138 mEq/L), n (%)^§^	1716 (20.0)	3805 (23.1)	324 (29.3)
Grade 3 or 4 (<130 mEq/L), n (%)^§^	34 (0.4)	571 (3.5)	72 (6.5)

ASM, antiseizure medication; 95% CI, 95% confidence interval.

^∗^One-way ANOVA; p < 0.001, post-hoc Scheffé test; ^†^p < 0.001 versus pediatric group, ^‡^p < 0.005 versus adult group.

^§^χ^2^ test; p < 0.001.

The mean serum sodium level was significantly lower in the older adult group than in the pediatric (Scheffé test, p < 0.001) and adult groups (p < 0.005). Also, the older adult group had a higher prevalence of moderate-severe hyponatremia (serum sodium <130 mEq/L) than the pediatric and adult groups (χ^2^ test, p < 0.001).

### 3.2 Incidence rate and incidence trends of hyponatremia from 2006 to 2020

**Fig. 1:**
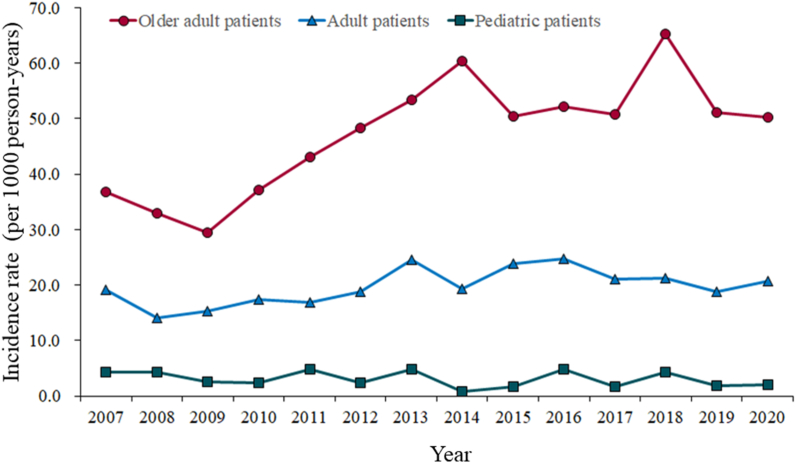
Annual incidence rate of moderate-severe hyponatremia from 2007 to 2020.

During the 15-year study period, 677 patients with a sodium level of less than 130 mEq/L had symptoms such as fatigue, headache, vomiting, anorexia, or coma. Among these patients, 39 (5.7%) were hospitalized for treatment of hyponatremia. Fig. 1 shows the incidence rate of hyponatremia in the three age groups. The mean incidence of hyponatremia per 1000 person-years was 3.1 (95% CI, 2.2–3.9) in the pediatric group, 19.8 (95% CI, 18.7–20.9) in the adult group, and 50.4 (95% CI, 43.3–57.5) in the older adult group. The group of older adults had a higher incidence of hyponatremia, and the rate increased markedly over the study period from 36.8 in 2007 to 58.5 in 2020. In contrast, the incidence rate of hyponatremia in the adult group remained almost unchanged (19.2 in 2007 and 20.7 in 2020). In 2007 and 2020, the incidence was lowest in the pediatric group and decreased over the study period (from 4.4 in 2007 to 2.0 in 2020). In the 15-year study period, the total number of incidences of hyponatremia in the four seasons were as follows: spring, 164; summer, 207; autumn, 170; and winter, 136.

**Fig. 2:**
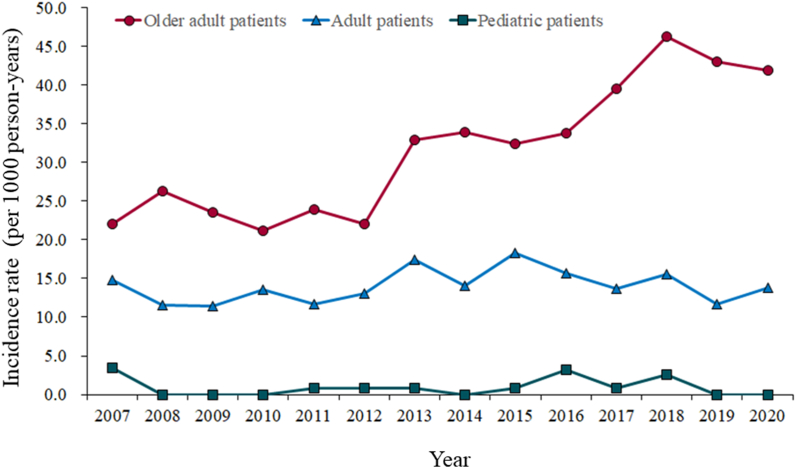
Annual incidence rate of carbamazepine-related hyponatremia from 2007 to 2020.

Furthermore, we evaluated the annual incidence rate of carbamazepine-related hyponatremia in the three age groups (Fig. 2). In the group of older adults, the incidence rate increased by about two-fold from 22.1 in 2007 to 41.8 in 2020, but no apparent change was observed in the pediatric and adult groups.

### 3.3 Risk factors for hyponatremia

Table 2 shows the results of the univariate analysis. Concomitant use of phenytoin, phenobarbital, and benzodiazepines was a risk factor for moderate-severe hyponatremia across the three age groups. However, several drugs had a different influence on the risk of hyponatremia in pediatric and adult/older adult patients. In the adult and older adult groups, use of carbamazepine and antipsychotics were significant risk factors for moderate-severe hyponatremia. Pediatric patients treated with carbamazepine tended to have an increased risk of hyponatremia, but the risk was not significantly higher than in pediatric patients not treated with this drug. Concomitant use of valproate was a risk factor in pediatric patients but not in adult or older adult patients. Also, although 161 pediatric patients in our cohort received antipsychotics, no cases of moderate-severe hyponatremia occurred in this group.

**Table 2:** Univariate analysis of factors that increase the risk of moderate-severe hyponatremia (serum sodium level <130 mEq/L).

	Pediatric patients (0–15 years)			Adult patients (16–64 years)			Older adult patients (≥65 years)		
	No moderate-severe hyponatremia (n = 8564)	moderate-severe hyponatremia (n = 34)	*P* value	No moderate-severe hyponatremia (n = 15,905)	Moderate-severe hyponatremia (n = 571)	*P* value	No moderate-severe hyponatremia (n = 1033)	Moderate-severe hyponatremia (n = 72)	*P* value
Age, mean ± SD, y	7.4 ± 0.05	6.8 ± 0.9	NS	32.7 ± 0.1	40.7 ± 0.5	<0.001	72.3 ± 0.2	74.4 ± 0.7	<0.01
Sex, n (%), female	3671 (42.9)	16 (47.1)	NS	7372 (46.4)	247 (43.3)	NS	454 (43.9)	26 (36.1)	NS
Concomitant drugs, n (%)									
Valproate	3413 (39.9)	20 (58.8)	<0.05	5875 (36.9)	231 (40.5)	NS	201 (19.5)	15 (20.8)	NS
Carbamazepine	1682 (19.6)	10 (29.4)	NS	4820 (30.3)	417 (73.0)	<0.001	261 (25.3)	42 (58.3)	<0.001
Phenytoin	438 (5.1)	6 (17.6)	<0.001	2922 (18.4)	142 (24.9)	<0.001	195 (18.9)	23 (31.9)	<0.01
Phenobarbital	620 (7.2)	9 (26.5)	<0.001	2497 (15.7)	146 (25.6)	<0.001	167 (16.2)	19 (26.4)	<0.05
Zonisamide	1130 (13.2)	6 (17.6)	NS	1505 (9.5)	36 (6.3)	<0.05	44 (4.3)	3 (4.2)	NS
Benzodiazepines	1533 (17.9)	15 (44.1)	<0.001	3221 (20.3)	248 (43.4)	<0.001	105 (10.2)	14 (19.4)	<0.05
Lamotrigine	755 (8.8)	4 (11.8)	NS	1577 (9.9)	70 (12.3)	NS	43 (4.2)	2 (2.8)	NS
Levetiracetam	1129 (13.2)	3 (8.8)	NS	1881 (11.8)	71 (12.4)	NS	113 (10.9)	7 (9.7)	NS
Topiramate	444 (5.2)	0 (0.0)	–	442 (2.8)	15 (2.6)	NS	7 (0.7)	0 (0.0)	NS
Lacosamide	100 (1.2)	0 (0.0)	–	370 (2.3)	6 (1.1)	<0.05	56 (5.4)	2 (2.8)	NS
Perampanel	81 (0.9)	0 (0.0)	–	339 (2.1)	15 (2.6)	NS	12 (1.2)	0 (0.0)	NS
Antipsychotics	161 (1.9)	0 (0.0)	–	1038 (6.5)	147 (25.7)	<0.001	24 (2.3)	9 (12.5)	<0.001

In all three age groups, none of the second- and third-generation ASMs, i.e., lamotrigine, levetiracetam, topiramate, perampanel, and lacosamide, showed an increased risk of hyponatremia. Although 444 pediatric patients used topiramate, none of them developed moderate-severe hyponatremia.

Table 3 displays the results of the logistic regression analysis. The crude odds ratio was about two-fold higher in children taking phenytoin or phenobarbital than in adults and older adults. In addition, pediatric patients taking valproate had a two-fold increased risk of hyponatremia, but this ASM had little influence on the risk in adult and older adult patients.

**Table 3:** Logistic regression analysis of risk factors for moderate-severe hyponatremia (serum sodium level <130 mEq/L).

	All patients (N = 26,180)		Pediatric patients (0–15 years) (n = 8598)		Adult patients (16–64 years) (n = 16,476)		Older adult patients (≥65 years) (n = 1105)	
	Crude odds ratio [95% CI]	*P* value	Crude odds ratio [95% CI]	*P* value	Crude odds ratio [95% CI]	*P* value	Crude odds ratio [95% CI]	*P* value
Concomitant drugs								
Valproate	1.1 [0.9–1.3]	NS	2.2 [1.1–4.3]	<0.05	1.2 [0.98–1.4]	NS	1.1 [0.6–2.0]	NS
Carbamazepine	6.3 [5.3–7.4]	<0.001	1.7 [0.8–3.6]	NS	6.3 [5.2–7.5]	<0.001	4.1 [2.5–6.8]	<0.001
Phenytoin	2.1 [1.7–2.5]	<0.001	4.0 [1.6–9.7]	<0.001	1.5 [1.2–1.8]	<0.001	2.0 [1.2–3.4]	<0.01
Phenobarbital	2.3 [2.0–2.8]	<0.001	4.6 [2.1–9.9]	<0.001	1.8 [1.2–1.8]	<0.001	1.9 [1.1–3.2]	<0.05
Zonisamide	0.61 [0.45–0.83]	<0.005	1.4 [0.6–3.4]	NS	0.64 [0.46–0.91]	<0.001	0.98 [0.30–3.3]	NS
Benzodiazepines	2.9 [2.5–3.4]	<0.001	3.6 [1.8–7.1]	<0.001	3.0 [2.6–3.6]	<0.001	2.1 [1.2–4.0]	<0.05
Antipsychotics	5.9 [4.9–7.2]	<0.001	–	–	5.0 [4.1–6.1]	<0.001	6.0 [2.7–13.4]	<0.001
Number of drugs								
No use (reference)	1.0		1.0		1.0		1.0	
1 drug	2.9 [1.9–4.4]	<0.001	2.8 [1.2–6.6]	<0.001	5.9 [4.3–8.2]	<0.001	3.5 [1.8–6.9]	<0.001
2 drugs	6.5 [4.4–9.6]	<0.001	6.5 [2.5–16.8]	<0.001	13.1 [9.5–18.0]	<0.001	6.1 [3.0–12.6]	<0.001
3 drugs	10.6 [7.1–15.8]	<0.001	11.7 [2.5–55.1]	<0.001	14.4 [9.9–20.9]	<0.001	9.8 [4.2–23.2]	<0.001
>4 drugs	13.4 [8.8–20.3]	<0.001	185.9 [17.6–1960.3]	<0.001	23.9 [13.1–43.6]	<0.001	15.4 [3.7–64.9]	<0.001

The univariate analysis identified the following five drugs as hyponatremia-inducing drugs: carbamazepine, phenytoin, phenobarbital, benzodiazepines, and antipsychotics. In all three age groups, receiving a higher number of hyponatremia-inducing drugs was associated with an increased risk of moderate-severe hyponatremia. However, because only one pediatric patient received four hyponatremia-inducing drugs, the 95% confidence intervals in the overall and pediatric groups were very large.

### 3.4 Influence of ASM dose on the occurrence of hyponatremia

Table S1 shows the relationship between the incidence of hyponatremia and ASM dose. In this analysis, patients were classified into three dose groups: no use (reference category), less than the 75th percentile dose, and greater than or equal to the 75th percentile dose. Because adult and older adult patients had common risk factors according to the logistic regression analysis (Table 3), we combined these two groups. In pediatric patients, benzodiazepines were the only ASM with a dose-related increased risk of hyponatremia. In adult and older adult patients, carbamazepine, benzodiazepine, and antipsychotics were dose dependently associated with an increased risk of hyponatremia. Although valproate, phenytoin, and phenobarbital tended to increase the risk of hyponatremia, they did not show a dose-effect relationship.

### 3.5 Multiple logistic regression analysis of risk factors for hyponatremia

Last, we built multiple logistic regression models. As mentioned above, we used the covariates that were significant in the univariate analysis. The final models included the dose effect of carbamazepine, benzodiazepine, and antipsychotics (see Table S1). In adult patients, use of valproate was included as a covariate because it influenced serum sodium levels.

Table 4 shows the results of the multiple regression analysis in pediatric patients. Concomitant use of valproate, carbamazepine, phenytoin, and phenobarbital was found to be a significant risk factor for moderate-severe hyponatremia (serum sodium level <130 mEq/L). Also, the odds ratio was 4.6-fold higher in pediatric patients with a daily diazepam dose greater than 0.25 mg/kg than in those not treated with benzodiazepines. Infant patients (age, <1 year) had a 3.5-fold higher incidence of hyponatremia events than children aged 1 year or older.

**Table 4:** Multiple logistic regression analysis of risk factors for moderate-severe hyponatremia (serum sodium level <130 mEq/L) in pediatric patients.

Risk factor	AOR	[95% CI]	*P* value
Age			
Infants (age, <1 year)	3.54	[1.31–9.54]	<0.05
Sex (female = 1)	1.17	[0.59–2.32]	NS
Benzodiazepine (diazepam)			
No use∗	1.0		<0.005
<0.25 mg/kg/day	2.28	[1.04–4.98]	<0.05
≥0.25 mg/kg/day	4.62	[1.68–12.7]	<0.005
Concomitant ASM			
Valproate	2.50	[1.22–5.14]	<0.001
Carbamazepine	2.47	[1.14–5.36]	<0.001
Phenytoin	3.56	[1.41–8.96]	<0.001
Phenobarbital	3.55	[1.60–7.89]	<0.001

AOR, adjusted odds ratio; ASM, antiseizure medication; 95% CI, 95% confidence interval.

∗ Reference category.

In adult patients, higher age and concomitant use of valproate and phenobarbital were significantly associated with hyponatremia (Table 5). The model showed that carbamazepine, benzodiazepine, or antipsychotic-induced hyponatremia occurred in a dose-dependent manner. In particular, use of carbamazepine was the most important risk factor for hyponatremia. In contrast, concomitant use of zonisamide reduced the risk of hyponatremia.

**Table 5:** Multiple logistic regression analysis of risk factors for moderate-severe hyponatremia (serum sodium level <130 mEq/L) in adult patients (aged 16–64 years) and older adult patients (aged ≥65 years).

Risk factor	AOR	[95% CI]	*P* value
Age			
18–39 years∗	1.0		<0.001
40–64 years	2.48	[2.08–2.96]	<0.001
≥65 years	4.72	[3.55–6.29]	<0.001
Sex (female = 1)	0.95	[0.81–1.13]	NS
Carbamazepine			
No use∗	1.0		<0.001
<700 mg/day	4.10	[3.35–5.02]	<0.001
≥700 mg/day	9.42	[7.52–11.8]	<0.001
Benzodiazepine (diazepam)			
No use∗	1.0		<0.001
<10 mg/day	1.71	[1.39–2.11]	<0.001
≥10 mg/day	2.23	[1.75–2.84]	<0.001
Antipsychotics (chlorpromazine)			
No use∗	1.0		<0.001
<200 mg/day	2.64	[2.06–3.38]	<0.001
≥200 mg/day	4.72	[3.41–6.53]	<0.001
Concomitant ASM			
Valproate	1.71	[1.44–2.05]	<0.001
Phenytoin	0.98	[0.81–1.20]	NS
Phenobarbital	1.38	[1.13–1.69]	<0.005
Zonisamide	0.68	[0.48–0.95]	<0.05
Lacosamide	0.80	[0.39–1.64]	NS

AOR, adjusted odds ratio; ASM, antiseizure medication; CI, confidence interval.

∗ Reference category.

## 4 Discussion

We previously evaluated 14,620 adult epilepsy patients during a 12-year period (from 2006 to 2017). In the present study, we expanded this cohort and evaluated a 15-year period (from January 2006 to December 2020) and also included a pediatric sample [19]. In Japan, the third-generation ASMs perampanel and lacosamide were approved in 2016. Furthermore, several case reports have suggested that use of sodium channel blockers such as lamotrigine and lacosamide can induce hyponatremia [17,21]. However, the analyses of our large cohort showed that second- and third-generation ASMs have no significant influence on the risk of this event.

Although hyponatremia is a frequent problem associated with epilepsy, to our knowledge, the number of cases and annual incidence rates have not been previously reported. In our cohort, during the 15-year study period, the overall incidence remained unchanged in pediatric and adult patients but increased significantly in older adult patients. Not only did the group of older adults have a higher incidence of hyponatremia, but the number of older patients aged 75 years and over increased from 52 patients in 2006 to 116 patients in 2020. In Korea, the prevalence of epilepsy in older people increased about two-fold from 2009 to 2017, showing a strong increase after the age of 75 years [22]. Similarly, our cohort showed about a two-fold increase in the number of patients aged 75 years and older, which resulted in an increased incidence of hyponatremia.

Generally, higher age can lead to a reduced capacity for sodium retention. Chung et al. reported that patients with severe hyponatremia have a higher risk of developing dementia [23]. In contrast, successful treatment of hyponatremia is associated with a reduction in the length of the hospital stay and improvements in gait and mentation [24]. Thus, as the population ages and the number of older adults with epilepsy increases, it is becoming more important to prevent hyponatremia.

This large cohort study demonstrated that use of first-generation ASMs, such as carbamazepine, phenytoin, phenobarbital, valproate, and benzodiazepines, and antipsychotics is a major risk factor for development of hyponatremia. Among the ASMs, carbamazepine was the most important risk factor, confirming the results of previous studies [7,8,19]. In the kidney, carbamazepine can act on the collecting duct vasopressin V2 receptor (V2R) directly and upregulate aquaporin-2 (AQP2), resulting in excessive reabsorption of water [10]. In addition, phenytoin, phenobarbital, and carbamazepine have an enzyme-inducing effect, resulting in decreased thyroid hormone levels. Hypothyroidism can lead to an increase in the arginine vasopressin level [25], which may be associated with hyponatremia. However, hypothyroidism-induced hyponatremia is rather rare and occurs only in severe hypothyroidism and myxedema [26]. Generally, AMS-induced hypothyroidism is mild or moderate, so it is not able to contribute to the reduction of serum sodium concentrations. Although the mechanism remains unknown, we should carefully monitor sodium level in patients using poly-inducer regimens.

In all age groups, benzodiazepines increased the risk for hyponatremia in a dose-dependent manner. An analysis of adults aged 55 years and older in the population-based Rotterdam Study found that use of benzodiazepines was associated with hyponatremia and that participants who received both thiazide diuretics and benzodiazepines had more severe hyponatremia than those taking diuretics or benzodiazepines alone [27]. Our findings support these results. Although the mechanism of benzodiazepine-induced hyponatremia remains unclear, benzodiazepines are known to influence the neurotransmitter gamma-aminobutyric acid, which has been shown to interact with vasopressinergic neurons [28].

Although use of antipsychotics is assumed to rarely induce life-threatening hyponatremia, the evidence to support this assumption comes from case reports and a few observational studies [29]. Our large cohort clearly demonstrated that concomitant use of antipsychotics and ASMs was associated with an enhanced risk of moderate-severe hyponatremia. According to a Swedish population-based case-control study, patients being treated with first-generation antipsychotics are more likely to experience severe hyponatremia (adjusted odds ratio, 2.1) than those being treated with second-generation antipsychotics (adjusted odds ratio, 1.3) [30]. In this study, more than half the patients (60%) were using second-generation antipsychotics, and we found a dose-dependent increase in the risk of moderate-severe hyponatremia. High-dose antipsychotics with a high risk of causing dry mouth can induce water intoxication, which may increase the risk of hyponatremia. Also, Kim et al. reported that haloperidol acts as a V2R agonist in the kidney and upregulates AQP2. Antipsychotics can induce hyponatremia in the same manner as carbamazepine [10].

Altuntas, evaluated 1950 inpatients and found that the highest hyponatremia incidence was observed in summer [31]. Although we did not perform a statistical analysis of our respective data, we observed a similar trend in our epilepsy cohort. In general, the sweating rate increases in the summer, resulting in higher sodium loss. The carbonic anhydrase inhibitor zonisamide and topiramate can induce hypohidrosis, which may lead to decreased sodium loss in patients taking these ASMs. In our study, concomitant use of zonisamide reduced the risk of moderate-severe hyponatremia in adult and older adult patients, and none of the pediatric patients taking topiramate developed hyponatremia.

To our knowledge, no previous studies have investigated the incidence trends and risk factors for hyponatremia. Results from our large cohort showed that pediatric patients have a lower incidence rate of moderate-severe hyponatremia than adult and older adult patients. However, the logistic regression model showed very similar risk factors in pediatric patients as in adult and older adult patients. Also, among the pediatric patients, infants had a 3.5-fold higher risk, so careful attention should be paid to infants with infection, vomiting, or diarrhea.

In the present study, we demonstrated that ASM polypharmacy was associated with an increased risk of moderate-severe hyponatremia. Carbamazepine, phenytoin, phenobarbital, valproate, benzodiazepines, and antipsychotics can induce hyponatremia. When a patient is taking several ASMs, physicians should consider reducing multiple risk factors. Further studies are needed to evaluate the influence of ASM regimens on blood arginine vasopressin and urine AQP2 levels.

A strength of the present study is the large sample size (including a pediatric population), which increases the statistical power and reduces the risk of estimation errors. However, the study also had several limitations. Because of the retrospective design, we could not evaluate non-pharmacological factors, such as dietary habits, water intake, hypothyroidism, hypopituitarism, diarrhea, and vomiting. Also, we did not evaluate the effect of oxcarbazepine, eslicarbazepine, tricyclic antidepressants, or selective serotonin reuptake inhibitors, all of which may be associated with hyponatremia. Furthermore, most researchers define hyponatremia as a serum sodium level of less than 135 mEq/L, making it difficult to compare other studies with ours (which used <130 mEq/L as the cutoff). Thus, further studies are needed to confirm the details of clinical assessments in patients with hyponatremia. Hyponatremia is associated with cognitive decline, but our study did not include tests of cognitive function. In older adults, the prescription of second- or third-generation ASMs can decrease the incidence of hyponatremia, but these drugs are relatively expensive; therefore, we plan to perform a cost-effectiveness analysis in the future.

In conclusion, the use of first-generation ASMs and antipsychotics and combinations of these drugs is a major risk factor for moderate-severe hyponatremia. Because the population of patients with epilepsy is currently aging, hyponatremia will become an increasingly important concern in clinical settings. Our findings can contribute to minimizing the risk of hyponatremia in epilepsy patients.

## Author contribution statement

Yoshiaki Yamamoto: Conceived and designed the experiments; Performed the experiments; Analyzed and interpreted the data; Wrote the paper.

Akiko Ohta: Contributed reagents, materials, analysis tools or data.

Naotaka Usui; Katsumi Imai; Yoshiyuki Kagawa; Yukitoshi Takahashi: Conceived and designed the experiments; Analyzed and interpreted the data.

## Data availability statement

The authors are unable or have chosen not to specify which data has been used.

## Declaration of competing interest

The authors declare that they have no known competing financial interests or personal relationships that could have appeared to influence the work reported in this paper.
