# Supplementary material for: Incidence trends and risk factors for hyponatremia in epilepsy patients: A large-scale real-world data study
Source: Heliyon. 2023 Jul 26;9(8):e18721. doi: 10.1016/j.heliyon.2023.e18721 (PMC10404753; doi:10.1016/j.heliyon.2023.e18721)
Supplement: Multimedia component 1 [file mmc1.doc]

Table S1 Relation between medication doses and incidence of severe hyponatremia (serum sodium level < 130 mEq/L)

|  | Pediatric patients  (0-15 years) | | | |  | Adult and older adult patients  (≥ 16 years) | | | |  |
| --- | --- | --- | --- | --- | --- | --- | --- | --- | --- | --- |
|  | Antiseizure medications | Dose  (mg/kg/day) | Crude odds ratio  [95% CI] | *P* value |  | Antiseizure medications | Dose  (mg/day) | Crude odds ratio  [95% CI] | *P* value |  |
|  | Phenytoin | No use | 1.0 | < 0.001 |  | Phenytoin | No use | 1.0 | < 0.001 |  |
|  |  | < 7.5 | 0.86 [0.12-6.31] | NS |  |  | < 300 | 1.74 [1.43-2.12] | < 0.001 |  |
|  |  | ≥ 7.5 | 14.7 [5.55-38.7] | < 0.001 |  |  | ≥ 300 | 2.07 [1.31-3.28] | NS |  |
|  | Phenobarbital | No use | 1.0 | < 0.001 |  | Phenobarbital | No use | 1.0 | NS |  |
|  |  | < 5 | 4.89 [2.10-11.4] | < 0.001 |  |  | < 100 | 1.98 [1.62-2.40] | < 0.001 |  |
|  |  | ≥ 5 | 3.88 [0.91-16.5] | NS |  |  | ≥ 100 | 1.43 [0.98-2.10] | NS |  |
|  | Carbamazepine | No use | 1.0 | < 0.001 |  | Carbamazepine | No use | 1.0 | < 0.001 |  |
|  |  | < 15 | 0.63 [0.19-2.11] | NS |  |  | < 700 | 4.01 [3.36-4.94] | < 0.001 |  |
|  |  | ≥ 15 | 6.20 [2.65-14.5] | < 0.001 |  |  | ≥ 700 | 11.4 [9.26-14.0] | < 0.001 |  |
|  | Benzodiazepine | No use | 1.0 | < 0.001 |  | Benzodiazepine | No use | 1.0 | < 0.001 |  |
|  |  | < 0.2 | 2.72 [1.19-6.23] | < 0.001 |  |  | < 10 | 2.28 [1.87-2.77] | < 0.001 |  |
|  |  | ≥ 0.2 | 5.98 [2.50-14.3] | < 0.001 |  |  | ≥ 10 | 3.79 [3.05-4.72] | < 0.001 |  |
|  | Valproate | No use | 1.0 | < 0.05 |  | Antipsychotics | No use | 1.0 | < 0.001 |  |
|  |  | < 30 | 1.94 [0.94-4.02] | NS |  |  | < 200 | 3.85 [3.06-4.84] | < 0.001 |  |
|  |  | ≥ 30 | 3.25 [1.16-9.04] | < 0.05 |  |  | ≥ 200 | 7.67 [5.71-10.0] | < 0.001 |  |

In univariate logistic regression analysis, *no use* was set at the reference category.

95% CI, 95% confidence interval
